# Modeling Contact Tracing Strategies for COVID-19 in the Context of Relaxed Physical Distancing Measures

**DOI:** 10.1001/jamanetworkopen.2020.19217

**Published:** 2020-08-21

**Authors:** Alyssa Bilinski, Farzad Mostashari, Joshua A. Salomon

**Affiliations:** 1 Interfaculty Initiative in Health Policy, Graduate School of Arts and Sciences, Harvard University, Cambridge, Massachusetts; 2 Aledade Inc, Bethesda, Maryland; 3 Center for Health Policy and Center for Primary Care and Outcomes Research, Stanford University School of Medicine, Stanford, California

## Abstract

This mathematical modeling study examines the potential for contract tracing to reduce the spread of severe acute respiratory syndrome coronavirus 2 (SARS-CoV-2) in the context of reduced physical distancing under different assumptions for case detection, tracing, and quarantine efficacy.

## Introduction

Confirmed coronavirus disease 2019 (COVID-19) cases have increased in the United States following the relaxation of strong lockdown measures.^1^ Contact tracing, which entails identifying and monitoring people who have been in close contact with individuals with confirmed diagnoses and encouraging them to self-isolate and quarantine, is recommended as a key component of COVID-19 control strategies.^2,3,4^ We used a mathematical model to examine the potential for contact tracing to reduce the spread of severe acute respiratory syndrome coronavirus 2 (SARS-CoV-2) in the context of relaxed physical distancing, under different assumptions for case detection, tracing, and quarantine efficacy.

## Methods

**Figure 1.**
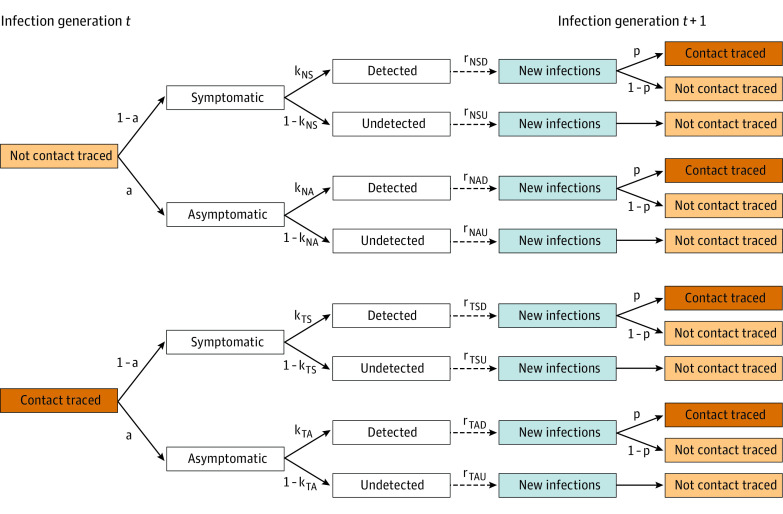
Model Structure and Parameters Parameter definitions: *a* is the fraction of infections that are asymptomatic; *k*, the fraction of infections that are detected; *r*, the number of secondary infections from each infection; and *p*, the fraction of cases that are successfully contact traced. For parameters indexed by subscripts: *T* is contact traced; *N*, not contact traced; *S*, symptomatic; *A*, asymptomatic; *D*, detected; and *U*, undetected. Parameter values are reported in the eAppendix in the Supplement.

In this mathematical modeling study, we developed a simple deterministic branching model of SARS-CoV-2 transmission (Figure 1; eAppendix in the Supplement). Individuals with infection transmit to others based on symptom status, detection of infection, and whether they are traced contacts of a known infected person. We varied the fraction of symptomatic infections detected in the community from 10% to 90%, the fraction of contacts successfully traced from 10% to 90%, the efficacy of isolation and quarantine among traced contacts from 30% to 90%, and whether testing included all identified contacts or only those with symptoms. We quantified the outcomes of contact tracing strategies as percentage reductions in the effective reproductive number (R_t_; the mean number of secondary infections from each infection) compared with a scenario without contact tracing. This measure is invariant to the starting R_t_, which enables comparisons across program scenarios that do not require calibration to specific epidemiological settings. All analyses were conducted using R version 4.0.2 (R Project for Statistical Computing). Because this study did not qualify as human participants research under the Common Rule definition, no institutional review board approval was sought.

## Results

**Figure 2.**
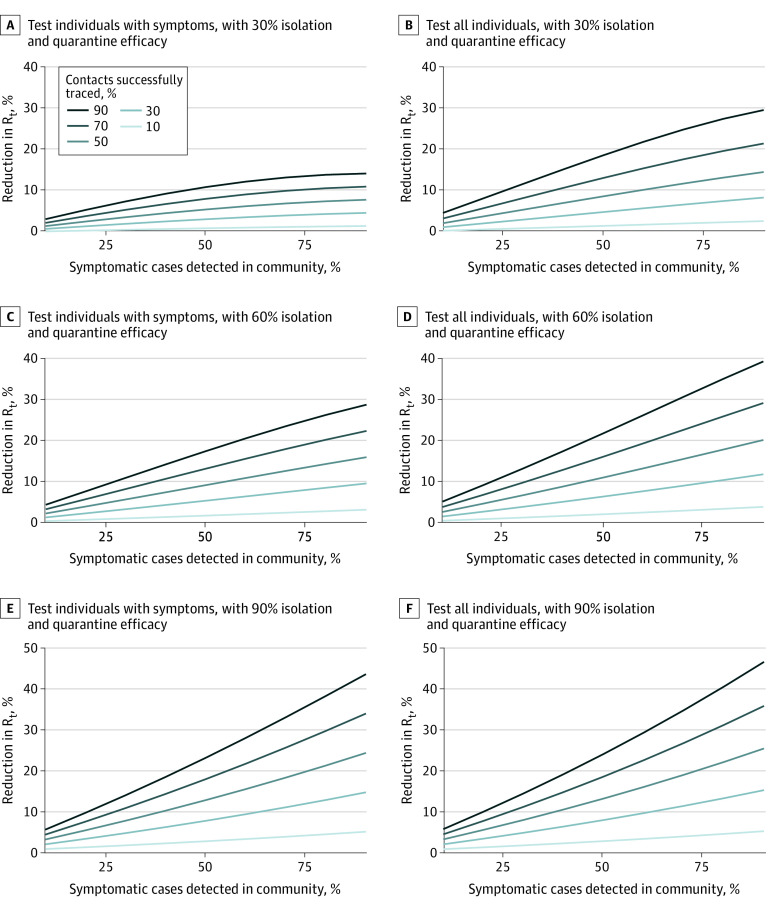
Reductions in the Effective Reproductive Number (R_t_) Associated With Contact Tracing Strategies Under Varying Assumptions Regarding Key Program Features Outcomes measured as percentage reductions in R_t_ in the contact tracing scenario relative to R_t_ without contact tracing. Isolation and quarantine efficacy refers to the level of reduction in transmission rates from traced, undetected contacts. Modeled estimates of relative reductions do not depend on current levels of R_t_.

When community detection of symptomatic index cases and tracing of contacts were less than 50%, simulated contract tracing programs did not reduce R_t_ by more than 10% (Figure 2). In scenarios with rates of detection and tracing that were both greater than 50%, testing asymptomatic contacts increased the program benefit by a median factor of 1.28 (range, 1.04-2.07), with a larger relative increase when isolation and quarantine efficacy were lower. The contact tracing scenario with the greatest benefit reduced R_t_ by 46%.

In sensitivity analyses, if the percentage of infections without symptoms was lower (20% vs base case of 40%), the benefit of contact tracing was greater, by a median factor of 1.22 (range, 1.04-1.41). In secondary analyses, we estimated the total combined benefit of improving case detection and contact tracing against the counterfactual of detecting only 20% of symptomatic infections and no contact tracing; the maximum combined benefit of tracing with higher detection was a 57% reduction in R_t_.

We calculated the degree to which contract tracing efforts could compensate for relaxed physical distancing and maintain R_t_ less than 1.0, which is the critical threshold needed for new infections to decline. As an example, if strict physical distancing decreased R_t_ from 2.5 to 0.9 and a contact tracing strategy could reduce R_t_ by 40%, containment remained possible if physical distancing measures were applied at only 52% of the effectiveness of strict measures.

## Discussion

To support efforts to control COVID-19, contact tracing must be implemented alongside prompt and extensive community case detection, and a high proportion of contacts must be reached. Similar to other models,^5,6^ our estimates imply that contact tracing could support partial relaxation of physical distancing measures but not a full return to levels of contact before lockdown.

The benefits of contact tracing depend substantially on adherence to isolation and quarantine among individuals who are traced, which could be enhanced through policy measures such as voluntary out-of-home accommodations, income replacement, and social supports. Prompt testing, diagnosis, and notification of individuals with infection are needed to ensure that contacts can be traced and quarantined early enough to prevent transmission. Testing contacts without symptoms could improve program benefits by identifying new cases to trace and potentially improving quarantine adherence.

Limitations of this analysis include lack of network or household structure or explicit consideration of high-risk venues. Nevertheless, by examining a range of scenarios that reflect key uncertainties and program features, we provided benchmarks to aid in developing evidence-based mitigation and containment strategies.

## Notes

Supplement. **eAppendix. **Supplementary Methods **eReferences.**
