# Supplementary material for: Modeling Contact Tracing Strategies for COVID-19 in the Context of Relaxed Physical Distancing Measures
Source: JAMA Netw Open. 2020 Aug 21;3(8):e2019217. doi: 10.1001/jamanetworkopen.2020.19217 (PMC7442925; doi:10.1001/jamanetworkopen.2020.19217)
Supplement: Supplement. — eAppendix. Supplementary Methods eReferences. [file jamanetwopen-3-e2019217-s001.pdf]

Supplementary Online Content

Bilinski A, Mostashari F, Salomon JA. Modeling contact tracing strategies for COVID-19 in the context of relaxed physical distancing measures. *JAMA Netw Open*. 2020;3(8):e2019217. doi:10.1001/jamanetworkopen.2020.19217

eAppendix. Supplementary Methods
eReferences.

This supplementary material has been provided by the authors to give readers additional information about their work.

© 2020 Bilinski A et al. *JAMA Network Open.*

eAppendix. Supplementary Methods

Table S1. Model parameter values.

**Parameter**	**Value**	**Notes**
Fraction of infections that are asymptomatic (*a*)	40%	Estimates vary across studies.[1–4] Alternative value of 20% examined in sensitivity analysis.
Fraction of cases detected		
Symptomatic, not contact traced (k_{NS})	varies	Values between 10% and 90% examined. Estimates on the fraction of symptomatic cases that are detected vary considerably across locations, due to testing capacity, epidemic intensity and other factors.[5]
Asymptomatic, not contact traced (k_{NA})	5%	Assumed to be negligible based on current US testing guidelines[6]
Symptomatic, contact traced (k_{TS})	90%	Assumption, reflecting referral to testing for traced contacts
Asymptomatic, contact traced (k_{TA})	90%	Assumption, reflecting referral to testing. Applies only in contact tracing strategies that include testing for asymptomatic people.
Number of secondary infections from each infection (*r*)	computed	See **Table S2** for details.
Fraction of cases successfully traced (*p*)	varies	Values between 10% and 90% examined. New York City results imply around 33% of contacts reached, adjusting for those who do not report any contacts.[7] Massachusetts has reported an increase from 40-50% to 90% of contacts reached.[8.] The UK has set a tracing target of 80%[9] and Santa Clara County, CA a target of 90%.[10]
Duration of infectiousness<br>Presymptomatic (d_P)<br>Symptomatic (d_S)<br>Asymptomatic (d_A)	 1.5 days<br>4 days<br>5.5 days	Durations inferred from temporal dynamics of viral shedding.[11]
Relative infectiousness of asymptomatic infection compared to symptomatic infection (v_A)	0.7	Estimates vary across studies (e.g. 1.0,[11] 0.66,[12] 0.5,[13] 0.25[14])
Relative infectiousness of presymptomatic infection compared to symptomatic infection	1	Inferred from studies indicating substantial presymptomatic transmission.[11,12]

© 2020 Bilinski A et al. *JAMA Network Open.*

(v_P)		
Relative number of secondary infections from detected infections compared to undetected infections (*q*)	0.5	Limited empirical data, rationale for reduced secondary transmission includes: potentially increased likelihood of adherence to self-isolation, targeting of confirmed cases for public health support.[15]
Average daily rate of transmission for symptomatic cases not traced (*b*)	Calibrated	Values calibrated to produce baseline R_t=1. Note that relative reductions in secondary infections, the primary outcome in this study, are invariant to the R_t level, so this calibration is only used in further application of primary results to estimate potential for containment with relaxed physical distancing.
Isolation and quarantine efficacy (*e*)	Varies	Values ranged from 30% to 90%. Isolation and quarantine efficacy is approximately the product of how much infectious time remains when the contact is notified, and the degree of adherence to isolation and quarantine measures. Estimates of adherence have ranged considerably in previous studies (0-94%)[16], including 70%[12] and 90%[17] in previous COVID-19 analyses. Remaining infectious time is difficult to measure, but likely less than 1.[17,18] A prior modeling study used efficacy estimates of 25% for a 'low-feasibility setting' and 75% for a 'high-feasibility setting.'[19]

Table S2. Estimation of secondary infections.

Category	Formula
Not contact traced, symptomatic, detected	$r_{NSD} = bd_Pv_P + bd_Sq$
Not contact traced, symptomatic, undetected	$r_{NSU} = bd_Pv_P + bd_S$
Not contact traced, asymptomatic, detected	$r_{NAD} = bv_Ad_Aq$
Not contact traced, asymptomatic, undetected	$r_{NAU} = bv_Ad_A$
Contact traced, symptomatic, detected	$r_{TSD} = (1\text{-}e)bd_Pv_P + (1\text{-}e)bd_Sq$
Contact traced, symptomatic, undetected	$r_{TSU} = (1\text{-}e)bd_Pv_P + (1\text{-}e)bd_S$
Contact traced, asymptomatic, detected	$r_{TAD} = (1\text{-}e)bv_Ad_Aq$
Contact traced, asymptomatic, undetected	$r_{TAU} = (1\text{-}e)bv_Ad_A$

© 2020 Bilinski A et al. *JAMA Network Open.*

eReferences

1. Oran DP, Topol EJ. Getting a handle on asymptomatic SARS-CoV-2 infection. Published April 20, 2020. Accessed July 21, 2020. https://www.scripps.edu/science-and-medicine/translational-institute/about/news/sarc-cov-2-infection/index.html
2. Gudbjartsson DF, Helgason A, Jonsson H, et al. Spread of SARS-CoV-2 in the Icelandic Population. *N Engl J Med*. 2020;382(24):2302-2315. doi:10.1056/NEJMoa2006100.
3. Lavezzo E, Franchin E, Ciavarella C, et al. Suppression of a SARS-CoV-2 outbreak in the Italian municipality of Vo'. *Nature*. Published online June 30, 2020. doi:10.1038/s41586-020-2488-1
4. Moriarty LF, Plucinski MM, Marston BJ, et al. Public health responses to COVID-19 outbreaks on cruise ships - worldwide, February-March 2020. *MMWR Morb Mortal Wkly Rep*. 2020;;69(12):347-352. doi:10.15585/mmwr.mm6912e3.
5. Russell T, Hellewell J, Abbott S, et al. Using a delay-adjusted case fatality ratio to estimate under-reporting. CMMID Repository. Published June 21, 2020. Accessed July 21, 2020. https://cmmid.github.io/topics/covid19/global_cfr_estimates.html
6. Pitzer VE, Chitwood M, Havumaki J, et al. The impact of changes in diagnostic testing practices on estimates of COVID-19 transmission in the United States. *medRxiv*. 2020.04.20.20073338. doi:10.1101/2020.04.20.20073338.
7. NYC Health + Hospitals. Test & Trace: Data. Accessed July 21, 2020. https://www.nychealthandhospitals.org/test-and-trace/data/
8. Mohl B. Contact tracing scaled way back: But Mukherjee predicts Mass. will face "second wave." *CommonWealt*h. July 7, 2020. Accessed July 21, 2020. https://commonwealthmagazine.org/health-care/contact-tracing-effort-scaled-way-back-2/
9. O'Dowd A. Covid-19: UK test and trace system still missing 80% target for reaching contacts. *BMJ*. 2020;370:m2875. doi:10.1136/bmj.m2875
10. Said C. Bay Area's contact tracers struggle amid coronavirus surge. *San Francisco Chronicle*. July 20, 2020. Accessed July 21, 2020. https://www.sfchronicle.com/health/article/Bay-Area-s-contact-tracers-struggle-amid-15419341.php
11. He X, Lau EHY, Wu P, et al. Temporal dynamics in viral shedding and transmissibility of COVID-19. *Nat Med*. 2020;26(5):672-675. doi:10.1038/s41591-020-0869-5.
12. Ferguson N, Laydon D, Nedjati-Gilani G, et al. Report 9: Impact of non-pharmaceutical interventions (NPIs) to reduce COVID19 mortality and healthcare demand. Imperial College London. Published March 16, 2020. doi:10.25561/77482.
13. Prem K, Liu Y, Russell TW, et al.; Centre for the Mathematical Modelling of Infectious Diseases COVID-19 Working Group, The effect of control strategies to reduce social mixing on outcomes of the COVID-19 epidemic in Wuhan, China: a modelling study. *Lancet Public Health*. 2020;5(5):e261-e270. doi:10.1016/S2468-2667(20)30073-6.
14. Zhao Z, Zhu YZ, Xu JW, et al. A mathematical model for estimating the age-specific transmissibility of a novel coronavirus. *medRxiv*. 2020.03.05.20031849. doi:10.1101/2020.03.05.20031849.
15. Barry E. In a crowded city, leaders struggle to separate the sick from the well. *New York Times* April 25, 2020. Accessed July 21, 2020. https://www.nytimes.com/2020/04/25/us/coronavirus-chelsea-massachusetts.html
16. Webster RK, Brooks SK, Smith LE, Woodland L, Wessely S, Rubin GJ. How to improve adherence with quarantine: rapid review of the evidence. *Public Health*. 2020;182:163-169. doi:10.1016/j.puhe.2020.03.007.
17. Kucharski AJ, Klepac P, Conlan AJK, et al.; CMMID COVID-19 working group. Effectiveness of isolation, testing, contact tracing, and physical distancing on reducing transmission of SARS-CoV-2 in

© 2020 Bilinski A et al. *JAMA Network Open.*

different settings: a mathematical modelling study. *Lancet Infect Dis*. 2020;S1473-3099(20)30457-6. doi:10.1016/S1473-3099(20)30457-6.
18. Ferretti L, Wymant C, Kendall M, Zhao L, Nurtay A, Abeler-Dörner L, Parker M, Bonsall D, Fraser C. Quantifying SARS-CoV-2 transmission suggests epidemic control with digital contact tracing. *Science*. 2020;368(6491):eabb6936. doi:10.1126/science.abb6936.
19. Peak CM, Kahn R, Grad YH et al. Comparative impact of individual quarantine vs. active monitoring of contacts for the mitigation of COVID-19: a modelling study. *medRxiv*. 2020.03.05.20031088. doi:10.1101/2020.03.05.20031088.

© 2020 Bilinski A et al. *JAMA Network Open.*
